# Supporting tumor therapy by exercise: boosting T cell immunity by myokines

**DOI:** 10.1038/s41392-022-01116-6

**Published:** 2022-08-22

**Authors:** Kristina Gebhardt, Karsten Krüger

**Affiliations:** Department of Exercise Physiology and Sports Therapy, Justus-Liebig-University of Giessen, Giessen, Germany

**Keywords:** Inflammation, Prognostic markers

In a recent study published in *Cancer Cell*, Kurz et al. reported how exercise induces inhibitory effects on tumor growth by immunological processes.^1^

The investigation of these mechanisms is particularly significant because lifestyle factors are becoming increasingly important as adjunctive therapy for cancer. For many tumor diseases, such as colorectal cancer and breast cancer, there are convincing data that physical activity has an impact on tumor growth, disease progression, mortality, and recurrence rates. The mechanisms are diverse and were initially attributed to indirect effects, such as increased metabolic turnover, anti-inflammatory effects of activity, regulatory functions on hormones, and antioxidative mechanisms. The study by Kurz et al. shows that exercise induces direct immunological effector functions via the release of myokines, which inhibits tumor growth.^1^

The evidence that exercise has a direct impact on cellular immunity to combat cancer is now convincing. The mobilizing and activating effects of each acute exercise bout on NK cells and T cells represent an essential process of the exercise-tumor interactions. Here, adrenergic mechanisms interact with activating signals through myokines.^2^ In response to regular training and increased cardiopulmonary fitness, long-term adaptations of the hematopoietic system are observed, which are reflected by shifts in proportions of T cell subpopulations towards more naive cells and less highly differentiated cell types. In addition, acute exercise and regular activity increase the proportion of regulatory T cells, contributing to a balanced immunological environment. From animal experiments, we could learn that regular running affects the metabolism of CD8^+^ cells and that cytotoxic T cells develop improved anti-tumor functions^3^.

During any form of physical activity, the skeletal muscle contracts and releases various signaling molecules that act in an autocrine, paracrine, and endocrine manner. The quality and quantity of the secreted molecules depend on the mode, duration, and intensity of the exercise. In addition to numerous metabolic effects, myokines exhibit multiple immunological functions. Many of these molecules, such as IL-6, engage in intense crosstalk with T cells, involving mobilization as well as emigration and infiltration of organs and tissues. This has also been shown for the myokine IL-15. IL-15 is expressed in skeletal muscle and released into circulation. Up to now, it is the only myokine promoting cytotoxic T cell survival.^4^

This is precisely where the study of Kurz et al. (2022) connects very well and fills a mechanistic gap. First, the study shows impressively that endurance training mobilizes T cells into the blood via increased adrenaline release. IL-15Ra cytotoxic T cells accumulate and infiltrate the tumor. This process is mediated by IL-15, but the study adds a central aspect of exercise immunology in the context of tumor diseases. The exact molecular signaling pathways of IL-15 in CD8 cells need to be elucidated in future studies. The model of pancreatic cancer used here has a special significance because it is a tumor disease with particularly high mortality. It seems important that a high infiltration with CD8^+^ cells after exercise was also shown in human tumors, whereby the human data support the results of the animal model. This does not seem to happen in all tumor types through exercise.

**Fig. 1:**
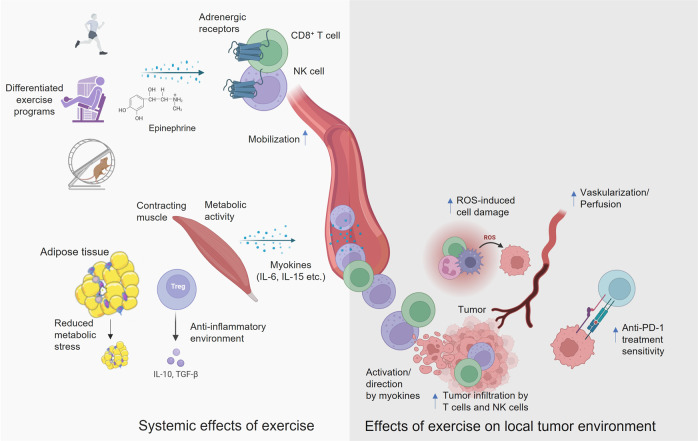
Systemic effects physical activity and associated local effects of exercise on the local tumor environment. Various modalities of exercise cause the release of epinephrine, which has a mobilizing effect on T cells and NK cells via adrenergic receptors. Myokines then activate and direct immune cells into the tumor, which can support therapy using checkpoint inhibitors (created by BioRender)

Another important aspect of the work is that physical activity represents only an adjuvant therapy, which is intended to support primary therapy and reduce side effects. Thus, there is often a lack of scientific work that considers physical activity accompanying pharmacological immunotherapies. Kurz et al. (2022) showed that in the context of the use of a PD-1 checkpoint inhibitor physical activity has a therapy-supportive effect. At this point, it would still be important to clarify the molecular mechanism by which type of exercise the tumor tissue is sensitized to PD-1 immunotherapy (Fig. 1).

The findings give great importance to the myokine IL-15, which is increased in circulation for 10–120 min after both endurance and strength training, regardless of age or training-status. Besides numerous metabolic effects, such as an improvement of glucose uptake and fatty acid uptake into the skeletal muscle, IL-15 also seems to have numerous immunological functions, such as activation of NK cells. The extent to which conclusions can be drawn here about IL-15 as an exercise mimetic must be shown by future studies. However, certainly physical activity can also make a significant contribution to tumor therapy via IL-15 release and the activation of CD8^+^ cells. If the holistic efficacy of exercise on tumor diseases is considered, patients benefit from numerous other effects. For exercise therapy, it was convincingly shown that it reduces cancer-related fatigue, combats cachexia, increases muscular and cardiovascular performance, enhances quality of life and many other significant factors for patients (Nadeau et al., 2018).^5^

From a clinical perspective, it will be important to investigate exercise recommendations more precisely. This includes the optimum modality, volume, duration, and intensity of exercise. The exercise protocols in the study by Kurz et al. (2022) are certainly a good start approaching differentiated exercise regimes in the mouse model. However, all these models certainly have a high barrier of transferability from murine models to the patient and training in clinical settings. For IL-15, systemic increases were also induced by strength or resistance training accompanied by an anabolic effect on muscle tissue. This will raise the question to what extent strength training also has similar effects in tumor therapy. Future studies should address the optimum exercise program for a considerable IL-15 release in patients including other current therapeutic approaches in oncology.
